# The Dynamics of Foreign Language Enjoyment: An Ecological Momentary Assessment

**DOI:** 10.3389/fpsyg.2020.01391

**Published:** 2020-07-14

**Authors:** Majid Elahi Shirvan, Tahereh Taherian, Elham Yazdanmehr

**Affiliations:** ^1^Department of English Language, University of Bojnord, Bojnord, Iran; ^2^Department of English Language, Yazd University, Yazd, Iran; ^3^Department of English Language, Attar Institute of Higher Education, Mashhad, Iran

**Keywords:** foreign language enjoyment, ecological momentary assessment, dynamics, enjoymeter, ecological sampling scheme

## Abstract

Following the recent shift from negative psychology to positive psychology, interest in foreign language enjoyment (FLE) has grown noticeably in second language acquisition. Given the fact that learners are “persons-in-context” and are not “ergodic ensembles,” the particular learner-context ecosystem goes through ongoing momentary changes with respect to individual differences like FLE. Ecological momentary assessment (EMA) contributes to our understanding of the dynamics of this ecosystem in terms of the interaction between individual learners and their learning environments. In this study, using a time-based sampling scheme of EMA, we explored the dynamism of different facets of FLE across different timescales including seconds, minutes, weeks, and months in a course of intermediate English as a foreign language. To do this, we applied open-ended interviews with two intermediate English language learners in a private English language institute across months, journals across weeks, enjoymeters across minutes, and the idiodynamic approach across seconds. Findings indicated that enjoyment in foreign language fluctuates in terms of a hierarchy of temporal scales, from moment-to-moment changes to the ones over months. The emerging patterns of enjoyment across different timescales in terms of the tenets of complex dynamic systems theory are discussed.

## Introduction

How emotions affect language learning has been highlighted more than ever before in recent years. Attention has also been drawn to what affects different learners experience in learning the language (Berdal-Masuy and Pairon, 2019; Dewaele, 2019; Dewaele et al., 2019b; MacIntyre et al., 2019). For long, copious attention has been drawn to learners’ negative emotions and attempts have been made to resist them. With the recent expansion of positive psychology in second language acquisition (SLA), it is increasingly known that resisting negative emotions is no guarantee for the replacement of positive emotions. Actually, recent advancement in emotion theory has shown that positive emotions such as enjoyment along with negative emotions including anxiety serve a variety of functions and stem from different kinds of experiences (MacIntyre and Gregersen, 2012; Fredrickson, 2013).

The significance of this changing trend lies in the fact that language learners experience negative emotions as well as positive emotions, including enjoyment, satisfaction, and perceived achievement to name but a few (Gkonou et al., 2017; MacIntyre and Vincze, 2017). Moreover, as described in the Broaden-and-Build Theory (Fredrickson, 2001, 2013), positive emotions would extend an individual’s thought-action resource and construct the social, physical, psychological, and intellectual base required for one’s current as well as prospective achievement. Therefore, showing positive emotions in second or foreign language learning might increase students’ awareness of linguistic input and understanding of language forms and enhance employment of different techniques for solving problems (Piechurska-Kuciel, 2017; Boudreau et al., 2018), which help to expand learners’ foreign or second language competence. Moreover, positive emotions can reduce the influence of negative emotions arising from problems of language learning, add to the flexibility from setbacks, and form social connections in class by running active approaches among other language learners and teachers (Dewaele and Alfawzan, 2018). This would further validate the change to positive emotions in SLA.

A quite relevant positive emotion in this respect is foreign language enjoyment (FLE). So far, this concept has been approached in research from different perspectives (Dewaele and MacIntyre, 2016; Li et al., 2018), including engagement with its origins and effects (Dewaele and Alfawzan, 2018; Dewaele et al., 2018) and how it correlates with anxiety in language learning (Dewaele and MacIntyre, 2014, 2016). In a recent critical review, Dewaele and Li (2020) invited researchers to investigate emotions involved in foreign language learning by developing original research designs in the light of their field as well as concepts and approaches adopted from similar disciplines. The present research, inspired by Dewaele and Li (2020), aimed to enrich the conceptualization and understanding of FLE system, and intended to shed light on the dynamics of FLE over time in the Iranian context, following an ecological momentary assessment (EMA).

Ecological momentary assessment has been primarily applied in clinical psychology and aimed to advance clinical psychology as a science and practice by illuminating the dynamics of behavior in authentic contexts (Shiffman et al., 2008). Ecological momentary assessment deals with the recurrent sampling of participants’ current experiences and behaviors in the real world and, thus, in their natural surroundings (Shiffman et al., 2008). In particular, EMA aims to reduce recall bias as far as possible, maximize ecological validity, and facilitate exploration of micro-processes that affect behavior in lifelike contexts. In fact, EMA studies address special events in subjects’ lives or evaluate individuals within intervals, often through random time sampling, with the help of technologies such as written diaries, phone calls, electronic diaries, and physiological sensors (Carson et al., 2010). The findings of this study can further our understanding of the structural constituents of FLE and reveal factors that contribute to the situated dynamic patterns of FLE. Considering this, the following research question was proposed in this study:

How do moments of FLE change under the influence of ecological factors in different timescales?

## Literature Review

### Foreign Language Enjoyment

Positive psychology emerged at the turning point of the present era as an independent discipline for research, marked by a refusal of a prevailing disease model of individuals’ performance in the field of psychology. It emphasizes mental problems and treatments, and it hopes to make sense of factors contributing to individuals’ psychological health and to help people and communities to develop successfully (Seligman and Csikszentmihalyi, 2000; Fredrickson, 2001). Positive psychology consists of three core ideas: positive institutions, positive individual traits, and positive subjective experience (Seligman and Csikszentmihalyi, 2000).

In 2014, the journal of Studies in Second Language Learning and Teaching published a special issue through which positive psychology entered SLA. In this journal, language experts such as Jean-Marc Dewaele, Sarah Mercer, and Peter MacIntyre recommended a change of research stream to positive emotions in the domain of SLA. This trend in the body of research on emotion is marked by a shift from a language anxiety-based trend in SLA research, in light of the Broaden-and-Build Theory of positive emotions (Li et al., 2018), and involves hints of the holistic perspective of negative and positive emotions together (Oxford, 2016). Ever since, copious research has addressed positive emotions in learning a second or a foreign language. The main positive emotions addressed have been enjoyment, love, flow, and pride (MacIntyre and Mercer, 2014; Pavelescu and Petric, 2018). Yet, enjoyment showed to be the most interesting to researchers in general and more recently in SLA in particular.

The simplest definition of FLE is marked by first highlighting how enjoyment differs from pleasure (Dewaele and MacIntyre, 2014). Enjoyment is defined as the pleasant feelings that originate from going beyond homeostatic boundaries as well as extending oneself to gain new experiences particularly when one encounters challenging tasks. On the contrary, pleasure is defined only as the pleasant feeling one develops when the homeostatic requirements (such as bodily comfort, sex, and hunger) are met hedonically (Dewaele and MacIntyre, 2016). In other words, enjoyment is described as “a sense of novelty and accomplishment” (Seligman and Csikszentmihalyi, 2000, p. 46) that facilitates long-lasting health and individual development (Seligman and Csikszentmihalyi, 2000).

Recent research in the FLE domain has shown an interest in dynamic mechanisms of the construct and its change within an individual or inside a group to gain a deeper understanding and to contribute to foreign language teaching and learning as well as assessment (e.g., Dewaele and Dewaele, 2017; Boudreau et al., 2018; Elahi Shirvan and Taherian, 2018). Such investigations have been enlightened by the dynamic system theory (DST), which has enriched second language acquisition, and have increasingly brought evidence that learner and teacher emotions change consistently and dynamically as a function of the interacting internal and external factors that might cause changes in the long run.

Along with the dynamic shift in second/foreign language learning, in light of DST, individual differences have been explored and emotions have been perceived as dynamic, dependent on context, and emergent (Ushioda, 2015; Larsen-Freeman, 2016). This emphasis on individual differences in SLA has been strengthened by the fact that language learners are not ergodic ensembles; that is, group statistics cannot be generalized to the individual (Lowie and Verspoor, 2019). Recent FLE research has thus been influenced by and enriched with the emerging research methods in DST (for a review of these methods see Hiver and Al-Hoorie, 2019). That is, via these methods, several studies have attempted to explore the dynamic aspects of FLE (e.g., Dewaele and Dewaele, 2017, 2020; Boudreau et al., 2018; Elahi Shirvan and Taherian, 2018; Elahi Shirvan and Talebzadeh, 2018a, b, 2020; De Ruiter et al., 2019; Talebzadeh et al., 2019).

Following a pseudo-longitudinal design, Dewaele and Dewaele (2017) addressed changes in FLE through time. To this aim, they adopted a dynamic approach. These researchers observed that learner-based and teacher-based variables minimally predicted FLE at the beginning and end of secondary education as compared to the middle phase. Elahi Shirvan and Taherian (2018) used a latent growth curve modeling approach to explore the growth and changing trends in 367 undergraduates’ FLE during a semester in a general English course. This research showed that, though the subjects’ FLE increased significantly within the semester, the significance of the intercept and slope variances for FLE showed heterogeneity of subjects’ growth in FLE during the study time. Besides, the initial level FLE did not manage to predict its growth within the semester.

Boudreau et al. (2018) employed an idiodynamic method to assess fluctuations in FLE and foreign language classroom anxiety (FLCA) in 10 Anglo-Canadian students in a second-by-second trend within 1 min. The values of the multiple FLE and FLCA correlation analyses in each subject showed a variation from positive to negative values. Moreover, high levels of FLE showed a momentary coincidence with low levels of FLCA. Yet, this association could entirely change a few seconds later. This could actually confirm the independence of FLE and FLCA dimensions. Similarly, Li et al. (2018) observed that FLE and FLCA both fluctuated severely, second by second, during Anglo-Canadian learners’ production of speech in French as their L2.

In a similar attempt, Elahi Shirvan and Talebzadeh (2018a) employed an idiodynamic method to see how enjoyment might fluctuate in conversations. To this aim, their research participants were provided with seven various topics graded in difficulty level. They were supposed to talk about these topics. The findings revealed intra- and inter-individual variation of enjoyment during the target conversations. Moreover, the participants talked about each topic and reported different levels of enjoyment. For example, while one topic may create a sense of enjoyment for one learner, the same topic might be less enjoyable for another learner.

Elahi Shirvan and Talebzadeh (2018b) also employed the idiodynamic method in another work of research on the non-verbal aspects of enjoyment. The aim was to see whether the possibility of enjoyment transfer in class ecology was true or not. This research revealed that on certain occasions enjoyment existed but was not perceivable, and, on other occasions, the non-verbal communication cues revealed enjoyment.

Still influenced by emotion contagion in psychology in another study, Talebzadeh et al. (2019) employed the idiodynamic method to investigate the dynamics and mechanisms of enjoyment contagion in five dyadic teacher–student interactions in a foreign language-learning course. This research concluded that automatic mimicry is the primary mechanism of enjoyment contagion involved in these interactions. Mimicry consists of facial expressions and gestures/postures including nodding, laughter, smiling, vocalic expressions, or leaning forward.

With a mixed-methods approach, Dewaele and MacIntyre (2019) looked into how learner-external and learner-internal variables affected FLE. Their research indicated that teacher-centered variables including teacher friendliness, attitudes toward teachers, and joking predicted FLE significantly.

Recently, De Ruiter et al. (2019) used Kohonen’s Self-Organizing technique to investigate the intra-individual FLE and FLCA process along with teachers’ degree of emotional support within the interactions of a teacher and two of his students in two pairs. This research revealed recurrent signs of teacher support as well as student anxiety and enjoyment. These signs would point to the self-organizing state of teacher–student interactions and the perception of students and teachers as dynamic systems in general. More recently, Elahi Shirvan and Talebzadeh (2020) applied a retrodictive qualitative modeling approach to explore the FLE signature dynamics. After identifying the learner archetypes of FLE through focus-group interviews with teachers and enquiring about their students’ enjoyment experiences, these researchers held in-depth interviews with a prototypical student from each archetype so as to unravel the trends and trajectories inducing a specific outcome or attractor state by tracking and investigating the dynamic events backward. The findings of this research added new insights to the dynamic trends that led to different FLE archetypes along with the adoptability of RQM to studies on enjoyment dynamics.

In the light of the aforementioned body of research, the trend seems to be moving toward more robustness realized through the development of psychometrically appropriate tools and application of triangulation to find more details of the dynamic aspect of FLE. The dominance of cross-sectional studies in the field reveals the need for more longitudinal designs and real-time exploration of FLE so as to make more comprehensive conclusions (Dewaele et al., 2019a). Furthermore, the new research directions that have appeared in the last few years have made it essential to evaluate the intrapersonal changes of students’ emotions as they actually emerge to better understand the dynamics and nuances of the complex nature of FLE system (De Ruiter et al., 2019). A salient property of this complex system is the momentary changes to L2 learner’s systems *in situ*. There are multiple methods being developed in the psychology of language learning to investigate this complex system. The focus in the majority of these new approaches has been on how the fluctuation within a univariate time series is mapped in time. For instance, the idiodynamic method that consists of self-ratings of fluctuations in such constructs as emotions (MacIntyre and Legatto, 2011) provides researchers with a chance to track momentary fluctuations of univariate time series. The retrodictive qualitative method identifies the end states and moves backward to see how developmental trajectories produce particular effects (Dörnyei, 2014).

Such methods are truly useful for investigating the development or variability of a unique construct such as FLE. Nevertheless, as there are different interacting components in the FLE system that define its dynamicity, certain methods are required that tap on the relationship between these components while they are emerging over time; a methodological tool is required to capture fluctuations in multivariate data. In spite of the methodological advancements observed in recent DST research, innovative approaches are thus still required to capture the detailed determining moments of change in an entire system as shown here with FLE. Such methods should ideally be capable of capturing how a phenomenon like FLE fluctuates and makes progress through time rather than as a static series of an ‘ordinary’ experience often gained in traditional single-shot measures. Ecological momentary assessment can be a typical innovative approach.

### Ecological Momentary Assessment

Ecological momentary assessment can be traced back to the experience sampling method (Csikszentmihalyi et al., 1977; Larson and Csikszentmihalyi, 1983), which aims to unravel phenomena as they occur in ordinary contexts of life (Stone et al., 1999). The designers maintained that the ecological value of physical, mental, social, or behavioral events that happen naturally would be significantly improved via data collection methods that obtain real-time information from actual contexts. Similarly, to realize the subjective and tentative nature of events as situated, the developers maintained that it was necessary for researchers to employ a method that could frequently assess the data through time. The three main properties of EMA are (1) assessments made as they occur in the field, (2) assessments involving many repeated observations, and (3) the required data assessed at a moment in time (Stone et al., 1999).

It is worth noting that the time-sampling approach in EMA can be associated with the concept of chronosystem in Bronfenbrenner’s (1989) nested ecosystems model. It refers to the changes occurring for an individual over his or her lifetime under the influence of events and experiences (Bronfenbrenner, 1989). Given this, in this study, we have regarded time as an important ecological setting in the emergence of language learners’ enjoyment. Additionally, ecological factors in this study refer to the factors based on which shifts of enjoyment take place in different timescales of their language learning.

Ecological momentary assessment-designed studies enjoy certain distinctive benefits that are largely absent in traditional, retrospective approaches. First of all, EMA studies enjoy a better resistance to pinpoint inaccuracies found in global self-reports that are used in such methods as retrodictive qualitative modeling. Many researchers observed that individuals either tend to dramatically underestimate or overestimate prior emotions, cognitions, and behaviors recalled through long periods of time (e.g., Thomas and Diener, 1990; Robinson and Clore, 2002). Furthermore, as documented, summaries of recollection are usually distorted by several reporting conditions during measurement, including a current mood state, a prevalent and salient event, or the most recent event (Brief et al., 1995). Collecting momentary data frequently, close in time to an actual experience, renders EMA an ideal approach to address the biases and distortions associated with retrospective reports (Smyth and Stone, 2003).

Second, EMA significantly increases the chance to trace meaningful intrapersonal variability that is generally lost in aggregate-level data (Beal and Weiss, 2003). Put it simply, EMA helps researchers to see how phenomena change across time rather than a fixed ordering of “typical” experience often obtained from nomothetic single shot measures.

Third, EMA helps researchers to scrutinize the contextual complexities as well as contingencies of everyday psychological constructs including emotions. The use of EMA has allowed researchers to show that immediate incidences of negative and positive emotions are the result of changing proximal stimuli (Zohar et al., 2003; Fisher and Noble, 2004), personality traits (Grandey et al., 2002), and perceptions of job properties (Fisher, 2002). Evidently, the experiential focus of EMA helps researchers to carefully explore and validate the dynamic processes of real emotional states from an ecological perspective, and it contributes to theoretical concepts (Scollon et al., 2003).

Considering the lack of studies using EMA to explore the dynamics of FLE in the literature of this construct, in this study, we maintain that the frequent assessment of the data through time can contribute to our understanding of the subjective nature of the participants’ moments of feeling enjoyment during different ecological timescales.

## Materials and Methods

### Participants and Context

This study was conducted in an intermediate course of English as a foreign language (EFL) in a private language institute in Mashhad, Iran. One of the problems with EMA is the considerable time commitment that it involves from the participants. Therefore, it is necessary to work with volunteer students in the class who could provide us with accurate data on the four timescales considered in this study.

In the class, we provided the students with a written statement of the aims of the study and the procedures of data collection. Two female students volunteered to participate in this study. They provided us with written consent for their volunteer participation in the project and they were told that they could leave the project whenever they were not at ease in any stage of the project. We met both at the end of one of the classroom sessions and provided them with more details regarding to their participation in the research project. We analyzed the data of two comparable participants. Both were from the same cultural and educational backgrounds (both Iranian) and of the same gender and level of competence (intermediate level) based on the Oxford Placement Test. They were both assigned pseudonyms (Sara and Sanaz) for ethical reasons. When the study was conducted, Sara was 21 years old and Sanaz was 20 years old.

### Data Collection

In this study, we applied a time-based sampling scheme of ecological momentary analysis. Inspired by Mercer’s (2015) study on the exploration of the dynamics of self, we generated the EMA data in this study to explore the participants’ moments of experiencing enjoyment on four timescales including seconds, minutes, weeks, and months using four different forms of data collection. To do this, we applied open-ended interviews across months, diaries across weeks, enjoy-meters across minutes, and the idiodynamic approach across seconds (see Table 1).

**TABLE 1:** Matrix of data collection.

**Level of FLE system**	**Timescale**	**Data collection tool**	**Spacing of data collection**
Micro-level FLE (Level 1)	Second	Idiodynamic method	Every second
In-class FLE (Level 2)	Minute	Enjoymeter	Every 5 min within one session
Weekly FLE (Level 3)	Week	Journal	Every week
Macro level of FLE (Level 4)	Month	Interviews	6 weeks apart

The micro-level form of data collection (Level 1) encompassed the application of the idiodynamic method (MacIntyre and Legatto, 2011). We invited the two participants to meet us out of their class time about the middle of the course. We asked them to do two different speaking tasks that took about 15 min. In the first task, they were supposed to introduce themselves to each other and ask each other about the recent activities they had enjoyed. We asked them to start with this task because its personal nature could make them feel at ease while they intended to know each other. They were supposed to carry out a more challenging activity in the second task that required them to argue about this prompt: “What impact has industrial changes in your country had on natural places?” We video recorded them while they worked on these speaking tasks. Having used the idiodynamic software program (Anion Variable Tester V2; MacIntyre and Legatto, 2011), we showed the recorded video of each participant’s involvement in the tasks to her so that she could watch and remember her moments of feeling enjoyment during the tasks. Next, we asked them to watch their recorded videos for a second time to rate their level of enjoyment during the two tasks via the use of a computer mouse on a scale ranging from –5 to +5. The software program then generated an output graph, in Excel format, of the fluctuations of both participants’ enjoyment level. Having considered this output graph as the guide for the interviews, we conducted semi-structured interview with each learner to gain deep information about the rises or falls in their enjoyment moments while they were completing the tasks.

We collected in-class enjoyment thermometer (enjoymeter) readings as our next layer of data (Level 2) across minutes in which the participants were asked to rate how much enjoyment they felt while using their English in the class at 5-min intervals. The enjoymeter was developed based on the longitudinal classroom studies undertaken by Gardner et al. (2004) and Waninge et al. (2014) in which an anxometer and motometer were used, respectively. To start with, we handed an A4 size sheet of paper with 20 enjoymeters to each participant. The enjoymeters were in the form of thermometer-shaped figures that ranged from 0, indicating the lowest enjoyment and 10 indicating the highest enjoyment points. We trained the participants in how to indicate their enjoyment level, which involved drawing a horizontal line on each enjoymeter every 5 min. We also audio-recorded each classroom session so that we could trace the fluctuations of each participant’s moments of feeling enjoyment. The participants were required to complete the enjoymeters every 5 min and add any comments regarding the levels of their enjoyment as well as the underlying factors contributing to those levels. This was conducted in three separate lessons that were spaced 3 weeks apart. We should mention that we decided to collect the first session of the enjoymeters data in the fourth week of the course because the class was more settled.

We collected our next level of data (Level 3) in the form of weekly journal entries. To provide the participants with chances of reflection in their journals, we provided them with the following prompt: “You should write about your moments of feeling enjoyment linked with your experiences of English language learning during the course.” They were not limited with any specific length. They both emailed us their written journals every week.

Finally, we collected a series of open-ended interviews as the macro-level (Level 4) in the participants’ first language, Persian. These interviews were spaced at almost three equal points, at the start, middle, and at the end of the 16-week semester. Before conducting the first interview, we asked the participants to prepare a self-description narrative of their language learning experiences during the course, in Persian, which were used as the basis for our first interview along with open-ended questions that we asked them in order to gain a holistic view of the participants’ moments of feeling enjoyment during the course.

### Data Analysis

We transcribed all of the collected data via MAXQDA software program. Then, through qualitative analysis, we coded all the transcribed data line-by-line so that we could take into account all aspects of the collected data in terms of any unexpected points in the analysis. Having conducted the rounds of coding, we combined or expanded the extracted codes until well-established categories emerged in terms of saturation level. The first and the second authors in this study did the coding process separately. That is, they both checked the extracted themes with each other, and they discussed any points of disagreement with each other to reach consensus on them. The inter-coder agreement was 90%, which is equal to the threshold suggested by Miles and Huberman (1994). Then, we examined the codes and categories with complex dynamic systems theory in mind and with a focus on the moment-to-moment changes of both participants’ enjoyment systems *in situ*. It should be noted that we did this process for each participant separately. We focused on each individual and developed her profile before moving on to the next. Finally, having analyzed the patterns of enjoyment in each level of data for each participant, we examined the possible interactions across each level of data for both participants.

### Sara’s Case

#### Sara’s Idiodynamic Data (Level 1)

**FIGURE 1:**
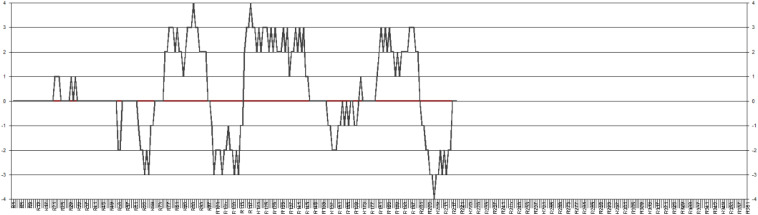
Sara’s idiodynamic graph.

Sara’s idiodynamic data were a combination of both moments of experiencing high and low levels of enjoyment (see Figure 1). Consistent with Dewaele and MacIntyre’s (2019) categorization of causes of emotions, our specific focus in this analysis was on the notion of different sources of FLE, including those associated with self, self–peer, self–teacher, self–peers–teacher (Dewaele and MacIntyre, 2019), as well as *FLE-Private* and *FLE-Social* dimensions (Dewaele and MacIntyre, 2016).

For Sara, factors contributing to her moments of low enjoyment in her idiodynamic data were revealed to be mainly bound to the *FLE-Private* dimension. More specifically, she went through moments of enjoyment under the factors related to her “self.” As seen in Figure 1, she went through low moments of enjoyment in the primary seconds of her interaction with Sanaz. Regarding these moments, she pointed out the following:

I was highly concerned about my grammar and structure, so I didn’t enjoy the primary seconds of the interaction. I know it was supposed to be a greeting type of conversation, but I was deep in my own thoughts on what I was to talk and how I would structure it.

As seen in Figure 1, in the middle of her interaction, she had some high moments of enjoyment. The emergence of these moments was still “self” bounded. With respect to these moments she reported the following:

Having gained the confidence in my speech I started enjoying the conversation as I found it pleasant. At the beginning of the second task, I had to think about the topic—that’s why my enjoyment was low in those seconds. Later, I liked the challenge in the second task. I mean at first it seemed difficult for me to deal with the idea of industrial effects on nature but later when I came up with the concept map and the right wording in my mind, I was so much engaged in the topic. It was like a joyful ride.

About her moments of low enjoyment in the final second of the interaction, she pointed to her being fatigued at the end of the interaction, as she was involved in the structuring of supporting ideas for the topics. This shows that throughout her interaction with Sanaz, almost all the dynamics of her moments of enjoyment were susceptible to a private zone of enjoyment with her “self” as the contributing ecological factor to these dynamic moments.

#### Sara’s Enjoymeters (Level 2)

**FIGURE 2:**
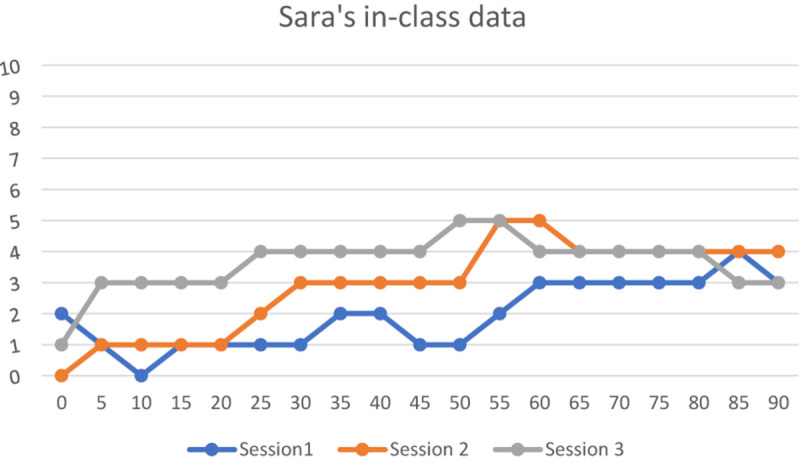
Sara’s in-class data (10 = high FLE; 0 = low FLE).

Sara’s enjoymeters indicated moments of high enjoyment during her in-class tasks as her range of enjoyment fluctuated between 1 and 5 in every 5-min period of the class activities. It should be noted that two of her single ratings were zero. As seen in Figure 2, none of her ratings were on the highest enjoyment extreme at 10. Unlike her idiodynamic, data which showed the contribution of only her “self” to her dynamic moments of enjoyment, her enjoymeter data indicated that other social factors could also pave the way for her enjoyment moments. That is, the traces of both *FLE-Private* and *FLE-Social* such as those related to “self,” “self–teacher,” “self–peers,” as well as “self–peers–teacher” could be seen in Sara’s enjoymeter data.

First of all, as it was dominant in her idiodynamic data, many episodes of her high enjoyment moments were also related to instances of her *FLE-Private*, such as her sense of creativity during the class activities. This sense was so enjoyable for her because she could make links between her background knowledge and the activities of the new session. For example, in the seventh session of the course (the second enjoymeter collection session), she experienced moments of high enjoyment in almost all the moments of the classroom because she could feel her creative power in the class. Regarding these moments, she explained the following:

Today, for the reading activity, we were supposed to read the first page of a short story and then predict the content of the next page. These moments were so enjoyable for me because I could make some good links with my background information of the movies I had watched and lots of stories I had read. Making a concept map of my thoughts on the page gave me a pleasant feeling as well. This joy was even higher when I could imagine myself as the author of the story thinking about how to go on with the rest of the story. This gave me food for thought.

As factors associated with “self–peers–teachers” contributing to her moments of feeling enjoyment, she referred to interacting with the same students and the teacher every week and having a group discussion with the peers.

The role of her teacher was noticeable in her moments of enjoyment. Some “self–teacher” related factors contributing to these moments were described:

(a) A positive teacher

According to Sara’s enjoymeter descriptions, her teacher’s praise, her personal attention, her recognition of the students’ efforts and encouragement provided enjoyable experiences for the students in almost all the moments of the three classroom sessions.

(b) Using humor in the class

Her moments of enjoyment were due to her teacher’s use of humor in the beginning of the seventh session and the middle of the 10th session of the course (the second and the third sessions of the enjoymeter data).

(c) Giving positive feedback

She appreciated her teacher’s constant use of appropriate feedback to the students in the class. Regarding the middle of the fourth session of the course (the first session of enjoymeter data), she pointed out “I felt very happy whenever I answered the teacher’s questions correctly and; in turn, she appreciated my responses.”

(d) Providing a pleasant and supportive atmosphere

She appreciated her teacher’s active role in making the class intriguing and engaging for her. She reported how well she could concentrate on the challenging activities in the class via the support of her teacher in helping her to engage with these activities.

On the other hand, some moments of low enjoyment were noted in her enjoymeter descriptions due to the lack of affective support on the part of her classmates. For example, with regard to the latter moments of the 10th session of the course (the second session of enjoymeter data collection, she explained that “During the discussion phase, I was so excited to share my opinions with the rest of the class, enjoying these moments; once I could not find the word “equipment,” and, instead, I used the Persian equivalent “tajhizat.” But everyone started laughing at me and I felt so embarrassed. This reduced the level of joy I went through some moments before.”

#### Sara’s Weekly Journal Data (Level 3)

Sara’s moments of enjoyment as reported in her weekly journals fluctuated each week, but, as is quite common with her idiodynamic and enjoymeter data, the ecological factors underpinning these weekly moments stemmed from a *FLE-Private* zone. During the course weeks, she was involved with her “self” concerns such as her speaking skills and her plans and strategies for learning English. Besides these “self” factors, like her in-class enjoyment moments, the “self–teacher” factors played a central role in the development of her *FLE-Social*.

During these weeks, she was involved with the ways she could improve her speaking skill. She referred to this in nine out of her 15 journal entries, sometimes with frustration (e.g., Journal entry 1 or 5), rendering her experienced moments of high anxiety, and, on other occasions, with hope (e.g., Journal entry 6), making her feel high in enjoyment. In her very first journal entry, Sara wrote the following:

In this course, I am so worried about my speaking skill. In the previous semesters we had few chances of improving our speaking sub-skills and, to be honest, I did not make that much effort. Now I feel very confused how I can improve my speaking skill. But I aimed to do my best on it. Being able to speak accurately and fluently is always a good vision for me in this course.

Throughout all of her journal and interview data, Sara repeatedly put emphasis on her belief in the development of this vision, as she pointed out above, and she was relatively positive that she could go through moments of enjoyment and improve her speaking skill as long as she had that vision in her mind. In her fourth journal entry, she wrote that “Whenever I see myself close to this vision of mine as a fluent speaker in the weekly sessions, I enjoy it more, but whenever I see it rather implausible, I go through moments of low enjoyment during the week.”

Also, she was involved with strategies, planning, and making to-do lists for the improvement of her English. For her, it was important to be organized and have a clear plan to be prepared for each weekly session. In her seventh journal entry, she noted the following:

To achieve in doing the assignments of this course, I have tried to have a good plan and follow the strategies, which have been introduced in the course books and by the teacher. I think this plan has made the process of language learning so enjoyable for me because I can concentrate well on what I do. Definitely, whenever I felt this sense of achievement during the week, I felt moments of high enjoyment. Sometimes feeling anxious that I might be behind the schedule, I went through moments of low enjoyment.

These considerable moments of anxiety and ambiguity could be seen in her journals underlying her unstable moments of experiencing enjoyment. She repeatedly expressed this uncertainty at various points. For example, in Week 6, before her oral presentation, she wrote the following:

This week, I have a presentation in English in front of all class. This is very stressful. For me, lecturing even in Persian is very difficult let alone speaking in English. I am thinking of the things that might happen during my lecture… What if my classmates feel my anxiety? What if I have wrong pronunciation? What if I make grammatical mistakes or forget words? It would be super embarrassing.

Then she switched to her plans, strategies, and made lists of what to do:

I like doing presentation when I’m prepared for it completely. So, I need some strategies to stick to in order to have a nice presentation. If I could prepare for the lecture, I can deeply enjoy it.

Similarly, in Week 9, directly before her speaking exam, a sharp decrease in her moments of enjoyment was observed. This was because she was anxious that she might not have a satisfactory performance in the exam.

Experiencing *FLE-Social* as reflected in her journal data, she referred to the positive role of her teacher in the emergence of her high-enjoyment moments. For example, in Week 7, she reported a serious moment in which the teacher gave her some advice on strategies she could use to improve her speaking skills. Afterward, she felt more positive and motivated. She once again wrote optimistically about a plan to work with a new strategy that, she hoped, would improve her speaking as well as receiving positive feedback from her teacher.

Likewise, the contribution of the teacher to Sara’s moments of enjoyment was tangible in her tenth week journal entry concerning her listening exam. At this point, she wrote the following:

Having talked to the teacher before the exam, everything went well, and I felt very relieved. I am enjoying my English now, as all the practices are worth all the efforts I have made up to now. This joy of leaning makes me think of the next stages of my learning English with more attention.

#### Sara’s Macro-Level Interview Data (Level 4)

In alignment with her experiences of enjoyment moments in the other ecological timescales, Sara’s interview data revealed the dominance of her private FLE. Her interview data indicated that her moments of enjoyment pivoted on her “self” concerns with her sense of progress in the course and her sense of ambiguity about her skills. In general, she felt that her moments of feeling enjoyment generally fluctuated over the semester. Regarding this fluctuation, in her end of the course interview she explained the following:

I can say that my moments of feeling enjoyment were not stable during the semester. This can be due to several factors, like the doubts I had about my own skills in the middle of the course, my future image of learning English, as well as my sense of satisfaction of, and pride in, of my efforts and the strategies I used to improve my English.

Despite the fluctuation observed in Sara’s moments of enjoyment during the course, the findings of the three interview sessions across the semester indicated that Sara’s trajectories of enjoyment moments had an increasing trend during the semester.

A noticeable feature of Sara’s data on the macro-level was her inclination to discuss her level of enjoyment with regard to specific aspects of English language such as listening and speaking. Much of the interview data reflected her journal entries regarding her speaking skills. She repeatedly noted how this area was new to her but how, with appropriate practice and strategies and teacher’s support, she could enjoy her improvement in her skills during the course. At the middle of the course interview, she felt a sense of progress in this area that has an important effect on her FLE.

It should be noted that these “self” concerns in Sara were intertwined with a “self–peer” factor that influenced the fluctuations in her moments of enjoyment. Mainly concerned with her speaking skill, in her first interview session, she referred to moments of low enjoyment by expressing how uncertain she felt about her speaking ability due to her peers’ judgment. She noted the following:

When speaking in the class, sometimes I was unsure of what I was saying. Then I noticed that I had some mistakes in my grammar; then I feel embarrassed in front of my classmates, and I could not enjoy the class.

Regarding her listening skills, in the first interview session, she also said the following:

At this moment, I enjoy my listening more than my speaking because I see less judgment on the part of my classmates. My good knowledge of vocabulary makes the meaning of the listening excerpts in the listening assignments so clear for. An enjoyable feeling comes to me.

The traces of “self–teacher” and “self–peer” teacher were found to be contributing to Sara’s high enjoyment at the end of the course. In her final interview, at the end of the course, she expressed more enjoyment as felt more confident in her speaking due to her teachers’ and classmates’ positive feedback and the supportive atmosphere of the classroom. She pointed out the following:

Thanks to the teachers’ affective support and the change in the atmosphere of the class in terms of positive feedback from my classmates as well as my own efforts in speaking assignments during the course, I am experiencing more moments of enjoyment than before because I feel more comfortable in talking and more fluent in speaking English now.

### Sanaz’s Case

#### Sanaz’s Idiodynamic Data (Level 1)

**FIGURE 3:**
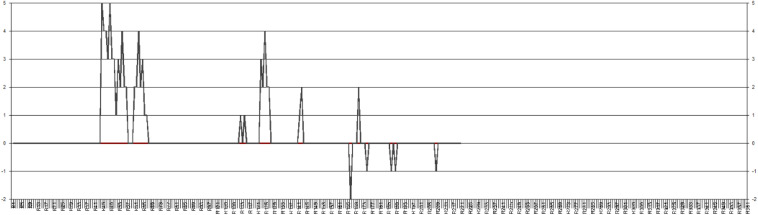
Sanaz’s idiodynamic graph.

Like Sara, Sanaz’s micro-level data is characterized by both high and low moments of feeling enjoyment, but she went through higher enjoyment moments while doing the tasks. As seen in Figure 3, her data did not vary within the full range of the scale, never going below –2, but extended up to +5 on the high enjoyment band following the overall positive trajectory of her enjoyment moments in her self-ratings. Also, an especially interesting feature of her idiodynamic data is that she changed frequently between the 0 and +5 bands, showing noticeable dynamism across the band of high enjoyment.

Quite different from the “self” oriented factors underlying Sara’s moments of enjoyment in her idiodynamic data, Sanaz’s moments of enjoyment were rooted in her “self–peer.” For instance, a pivotal factor contributing to Sanaz’s moments of enjoyment in her idiodynamic data was the enjoyment contagion emerging during her interaction with Sara. Regarding this contagion, she explained the following:

My moments of feeling enjoyment were all dependent on what feelings I could get from Sara. In the primary seconds of our interaction, I had no specific feeling because she seemed to be in herself, and didn’t look at me. Later, when she started to talk enthusiastically about the topic, I felt the joy in our interaction.

As seen in Figure 3, she went through some ups and downs in her moments of enjoyment in the high enjoyment band of her idiodynamic graph. With respect to these moments, she said the following:

These were the seconds when I had my preparation time to talk on the second topic; I expected Sara to keep her eye contact with me, but she was involved in her own thoughts, so I felt some drops in my enjoyment in these moments.

We should note that despite the differences in the sources of the ecological factors contributing to both participants’ moments of enjoyment, the *FLE-Private* was revealed to be a common domain of enjoyment for both. Like Sara, Sanaz went through a falling enjoyment trajectory at the end of her interaction. Trying not to be embarrassed before Sara, some traces of “self” related factors were revealed in her dynamic trajectory of enjoyment, as she was mentally loaded with the structure and wording of her sentences. She mentioned the following:

In the final seconds of our conversation, seeing Sara talking so passionately about the second topic, I didn’t want to feel embarrassed in front of her by making erroneous structures since the topic was a bit demanding. So, for some moments, I was in my own thoughts choosing the right words and grammar. This made me away from the joyful moments of the previous seconds.

#### Sanaz’s In-Class Data (Level 2)

**FIGURE 4:**
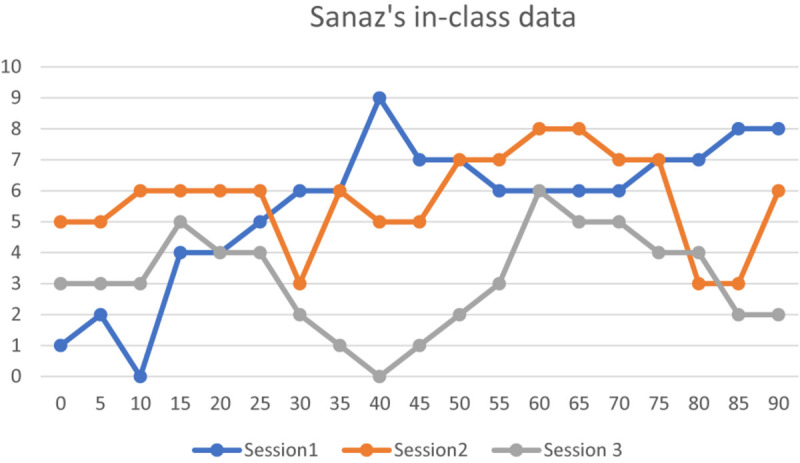
Sanaz’s in-class data (10 = high FLE; 0 = low FLE).

Sanaz’s enjoymeters dynamics also represented more variation in her enjoyment than Sara’s as it stretched across their full range. As seen in Figure 4, Sanaz’s trajectories of enjoyment in the first and the second sessions of the enjoyment data collection phase indicate moments of higher enjoyment than her trajectory in the third session. Quite like her idiodynamic data, her enjoymeter data highlighted the dominance of her *FLE-Social* in the dynamics of her in-class moments of enjoyment.

Among the factors associated with Sanaz’ *FLE-Social*, like what Sara experienced, the contributing role of the teacher to Sanaz’s enjoyment moments was considerable. For instance, in her enjoymeters descriptions related to her middle-class moments of the first and second sessions of the enjoymeters data collection, she referred to the encouraging role of her teacher in the development of her classroom performance. She noted that her moments of high enjoyment in the first session, especially in the middle of the session, were shaped by her teacher’s constant monitoring as well all as affective support of her and the other students in the class in doing the classroom activities.

Furthermore, like what was revealed in her idiodynamic data, another contributing factor to her moments of high enjoyment in the middle of the second session of enjoymeter data (the seventh session) was her frequent use of English with her peers. Sanaz regarded these moments as enjoyable due to her opportunities for classroom communication with her peers. She stated the following:

Generally, I enjoyed the class; there was a friendly atmosphere. My classmates were all open up. My classmates’ lecture was so funny. We had a nice group discussion about the Yalda night.

Susceptible to her peers’ judgment of her performance in the third session of the enjoymeters data collection phase (the 10th session of course), she went through moments of lower enjoyment than the previous two sessions. With respect to the moments, she maintained the following:

Today I was not that much ready for the classroom assignments. I was worried that I would be embarrassed in front of my classmates in case of my weak performance in responding to the teachers’ questions. I was deeply involved in these thoughts. So, in those moments in which I had a satisfactory performance, I felt that I could handle that session performance; so my enjoyment was high but in some moments I was still not sure about my performance. So, my enjoyment fell and it was mainly in the middle of the session.

Also, the outside class experiences such as social relations were a salient factor in the low levels of Sanaz’s enjoyment moments in the primary min of the second session of the enjoymeter data (the seventh session of the course). Regarding this, in her enjoymeter descriptions, she implied the following: “I had an argument with one of my best friends the day before. Considering this, in these moments I can’t enjoy my moments in the class.” This indicates that, despite the fact these experiences took place beyond the immediate setting of the classroom, they could overshadow the Sanaz’s in-class enjoyment moments.

#### Sanaz’s Weekly Journal Data (Level 3)

Consistent with the micro level and in-class timescale moments of enjoyment, Sanaz’s journal entries revealed her moments of *FLE-Social* under the influence of “self–peer” and “self–teacher” factors and in particular enjoyment contagion.

One of the things that characterized her journal data was the importance that she assigned her classmates and her teacher when writing about her sense of enjoyment in terms of her social contact with her classmates. She referred to these issues in 10 out of 13 journal entries. Sanaz expressed this influence directly in journal entry 12: “All people influence me—they influence how I feel and how I evaluate my skills in general.” This influence involved her classmates’ and her teacher’s feedback or opinion and social comparisons. For instance, the way her classmates felt in their classroom interactions or group work assignments contributed to her fluctuations in her sense of enjoyment. Regarding this, in her second journal entry, she noted that, “As long as my classmates feel positive in our group-work activities, I feel language learning can be an enjoyable process.”

In her fifth journal entry, she pointed out the following:

One of my group mates did not feel positive as I talked about the task to her because she was down; that made me feel down too. When I asked the reason for her low mood, she referred to an outside class accident for her.

Also, she highlighted the role of her teacher as a significant factor to her moments of feeling enjoyment. She referred to some factors such as the teacher’s non-threatening techniques and her efforts in providing motivation building strategies as well as promoting cooperation among learners.

In her seventh journal entry, she said, “Having done the reading assignment this week, I received the teacher’s positive feedback. This gave me a sense of pride, encouraging me to continue the other assignments with more focus.” Moreover, in her ninth journal entry, she wrote the following:

I really enjoyed my speaking when I saw the teacher’s smile while I was not sure about the structure I used in my speaking. It reminded me of the teacher’s statement in the previous session that what matters in the speaking time is our willingness to communicate and maintaining the flow of interaction.

On the other hand, like Sara’s private experiences of enjoyment, Sanaz went through some moments of private FLE due to a “self-”oriented factor. The dynamics of some of her enjoyment moments during each week were quite bound to her involvement with her past experiences of learning English. That is, her past experiences seemed to represent a heavy weight for her present feelings and she frequently compared her experiences in the present to her past. This, present in eight of the weekly journal entries, often gave her a positive sense of progress. For instance, she remarked, “Seeing my progress in English in this semester is so clear for me. Once I found learning English difficult, but it sounds easier and more enjoyable for me now” (Journal entry 8). Also, in her fourth journal entry, she wrote the following:

Compared to my past learning English, my attitude to this language has changed as well. Previously, I thought that I should try speaking or writing in English whenever I’m fully prepared for them, which made learning English stressful. Now, I’ve acquired this attitude that full mastery of structure and vocabulary is not needed to start a communication, but they emerge within the classroom communication.

#### Sanaz’s Macro-Level Interview Data (Level 4)

The moments of experiencing *FLE-Social* were notable in Sanaz’s interview data. One particularly interesting aspect, notable across the three interview sessions, was related to her dynamics of enjoyment moments with respect to the teacher’s and her peers’ roles, which fluctuated from interview to interview.

In her first interview session she said the following:

The teacher and the students in the class definitely play a pivotal role in how I might feel in the course. I know how I am dependent on their feelings. Once I felt the teacher was not satisfied with my writing assignment, it came to me that writing in English might not be as enjoyable as the other skills like reading and speaking.

The influence of the teacher in her feelings was also tangible in her second session interview:

I noticed that that writing in English can be as enjoyable as reading, and there is no difference in enjoyment level of the different skills, and I owe these moments of my enjoyment in doing writing assignments to my teacher who appreciated my writing performance.

Considering her overall enjoyment moments in the course, in her third interview she said the following:

Thanks to the teacher, my classmates’ overall attitude toward each other’s classroom activities was supportive and positive, which rendered the atmosphere of the class so enjoyable for me.

Like what Sara experienced as *FLE-Private*, the “self-”oriented factors of enjoyment were evident in her interview data. For example, she perceived her high enjoyment moments associated to her sense of self-satisfaction in her exam achievements. Regarding these post-exam moments of enjoyment, she said, “Success in exams of the course is enjoyable. It reminds me of my progress and change in my English ability.”

Furthermore, the interview data showed that Sanaz’s moments of enjoyment were not limited to her performance in the assignments of the course, but she went through further moments of enjoyment when she noticed how well she could take advantage of her English competence beyond the boundaries of her class assignments. Regarding this, in her third interview session she noted the following:

The practice of the videos with the video books in the course helped me try watching some movies at home by myself, an experience that I didn’t dare to do before but I found so enjoyable. I even write down the summaries of some of these movies and share it with my friends on social media.

## Discussion

The participants’ moments of experiencing enjoyment can be explained via the principles of broaden-and-build theory as a main theory in positive psychology (Fredrickson, 2001, 2013). For instance, as seen in her micro-level timescale data, Sara embraced the challenges in her tasks and the positive sense she felt as she was coping with these challenges provided her with the psychological base and sense of flow to accomplish the tasks. In other words, despite the fact that the challenges in the tasks seemed difficult for her when she started doing them, she did not quit the tasks; instead, her moments of going through enjoyment during the tasks extended her thought-action repertoires to accomplish them.

Moreover, as seen in her weekly journal data, her moments of enjoyment during the course are associated with her expansion of thought–action repertoires that are in line with previous research (Piechurska-Kuciel, 2017); these support the utilization of different problem solving techniques. At the beginning of the course, she was worried about her proficiency in the speaking activities, but her positive sense of being engaged in these activities helped her put more effort into her study in the course to improve her English proficiency. Besides, her belief in the construction of enjoyable moments in her speaking performance, as long as she worked hard, enabled her to enhance her mental resources by using appropriate strategies and plans to improve her speaking skill during the course. Importantly, Seligman and Csikszentmihalyi (2000) notion of enjoyment as a sense of novelty and source of personal development underpinned Sara’s moments of enjoyment. As seen in her enjoymeter data, the use of novel and various materials in the seventh session of the course enhanced her personal development and resulted in a sense of enjoyment for her.

On the other hand, as seen in Sanaz’s enjoymeter data, her moments of feeling enjoyment in the middle of the second session were intertwined with her construction of the social resources contributing to the establishment of a strong bond with her classmates. That is, via her socialization with her peers as social resources she experienced enjoyment moments in that session of the course, and this sense contributed to expanding her social bond with peers as well. Furthermore, Sanaz’s macro level timescale data indicated that she learned to expand her moments of enjoyment beyond those associated with her satisfaction in her exams. In fact, her moments of positive emotions during the exams enabled her to construct an intellectual base for her continuous willingness to use English by realizing the usefulness of English beyond her performance in the exams.

In addition, in alignment with Dewaele and Alfawzan (2018), language learners’ feelings toward each other played a pivotal role in the dynamics of moments of enjoyment. For instance, as indicated in Sanaz’s enjoymeters as well as her weekly journals, the emergence of her moments of enjoyment was highly dependent on her classmates’ affective approach toward her in the class. Their positive and negative feelings were reciprocated by her and, thus, made her experience both high and low enjoyment. This is what Elahi Shirvan et al. (2019) regarded as FLE contagion. Furthermore, quite consistent with the strong bond between the feelings of enjoyment and anxiety in learning a foreign language (Dewaele and MacIntyre, 2016, 2019; Elahi Shirvan and Talebzadeh, 2020), the findings indicated that learners’ related factors shaped their moments of high anxiety and, thus, low enjoyment, while teachers’ related factors provided them with high-enjoyment moments. In other words, when the participants were in their own “self” thoughts with regard to the choice of the right words and structure or when they felt anxious not to be embarrassed in front of their peers, they went through low-enjoyment moments. On the other hand, whenever they received their teacher’s support and positive feedback, they experienced high-enjoyment moments.

### Unique Ecological Moments of Enjoyment

In agreement with the previous research (Dewaele and Dewaele, 2017; Elahi Shirvan and Taherian, 2018; Elahi Shirvan and Talebzadeh, 2018a, b), all levels of timescale data showed that both participants’ moments of enjoyment during the course were heterogeneous. That is, they experienced both moments of high and low enjoyment. However, both participants’ sets of timescale data differ in content and dynamics influenced by ecological factors ranging from micro to macro level. Such divergences show the idiosyncrasies of individuals, the feelings they express and the latent complexity lying in each participant’s time of enjoyment, which in line with Lowie and Verspoor (2019), indicates that language learners are not ergodic ensembles. Three main differences can be seen in both sets of data: (1) differences in content and focus of FLE system; (2) differences in terms of the drivers of change in each learner’s moments of enjoyment; and (3) differences in numbers, the types and forms of dynamics.

### Differences in Content and Focus of the FLE System

Both learners differed strongly on the different timescales of FLE in terms of the content and focus of what they reported, but the more micro-level enjoyment moments have a more distinctive scope. Indeed, Sanaz’s content and focus of her dynamic system of enjoyment reflect the more social dimension of FLE (Dewaele and MacIntyre, 2016) such as her shared opinions and ideas with her interlocutors, classroom laugher, and pleasant relationships inside and outside of the class. In contrast, Sara’s FLE-system focus reflected the private dimension of FLE by experiencing joy when she felt pride, fun, or a sense of progress during the course.

At the micro level, the two participants’ trajectories of enjoyment could be divided into two dimensions of *FLE-Private* and *FLE-Social*. Sara was mainly involved with her “self” concerns of finding the right wording and structure in her conversation while Sanaz was heavily dependent on how Sara felt during the interaction. Despite the dominance of these two patterns of enjoyment moments of both participants in the other timescales, similarities were revealed between their moments of enjoyment. For instance, at the micro level, while keeping her eyes on Sara’s emotions, Sanaz in some moments felt less enjoyment than at other moments due to her mental loading of choosing the right words for the second topic that seemed more demanding for her.

Also, within the situated in-class time setting and weekly classroom sessions, while both participants went through moments of different, social vs. private, dimensions of enjoyment, their patterns of enjoyment emerged in some moments from a common source. For instance, the “self–teacher” factor was a common factor underpinning both Sara’s and Sanaz’s moments of enjoyment in these timescales.

At the macro level, Sanaz was more holistic while elaborating on her time of enjoyment and referred to her FLE on a broader social scope. Experiencing both *FLE-Social* and *FLE-Private*, she also made clear links between her moments of enjoyment under the influence of her teacher and her classmates, giving the impression of a more interactive system. In contrast, Sara expressed a tremendously more disintegrated, private FLE, primarily focusing on enjoyment to particular “self” concerns like the development of her speaking skill.

### Differences in Terms of the Drivers of Change in Each Learner’s System of Enjoyment Moments

Concerning what makes changes to each participant’s system of enjoyment, different factors can be involved individually. This shows that the main factors and the primary causes of change in a person’s FLE system cannot be regarded as universal, but they vary across individuals depending on their own unique characteristics. One such cause of change was the significant patterns among the systemic factors for each learner. For Sara, her moments of enjoyment were mainly driven by “self-”oriented ecological factors underpinning her *FLE-Private*. Furthermore, it was interesting that these “self-”oriented causes of change were not necessarily equal for each timescale of enjoyment. For instance, her mental involvement with the right wording and grammar was a constant “self-”oriented factor determining her moments of enjoyment, but the weight of this determining factor was overshadowed by other social factors like the positive influence of her teacher in the other timescales.

For Sanaz, the main drivers of change were clearly social and shaped by “self–peer,” “self–teacher,” and “self–peer–teacher” factors. These same drivers of change were apparent at different levels of her enjoyment system. In the idiodynamic data, for example, they are reflected in her strong focus on Sara’s reactions and, in the interview and journal data, in her affective responses to her experiences with others both inside (e.g., teacher and peers) and outside (e.g., friends) the classroom.

### Differences in Terms of Intra- and Inter-Variations of Enjoyment Moments

The findings of the study indicated that frequent assessment of the data through time could contribute to our understanding of the subjective nature of the participants’ moments of feeling enjoyment as situated in different ecological timescales. As seen in the findings, each timescale provided its unique affordances to the dynamics of each participant’s moments of enjoyment. For instance, for Sara, the underlying factors contributing to her moments of enjoyment were all bound to her “self” at the micro-level in terms of the seconds. However, in the in-class timescale, her moments of feeling enjoyment were shaped by the influence of social FLE factors associated with “self–teacher” and “self–peers–teacher.” This means that, even for the same individual learners, the patterns of enjoyment might emerge differently under the influence of different factors at each ecological timescale. Also, each ecological timescale contributed to the inter-individual variation in moments of enjoyment. As seen in the findings, the micro level timescale shaped the emergence of two different patterns of enjoyment for the two participants. That is, for Sara, her moments of enjoyment in the micro timescale were mainly *FLE-Private*, but those of Sanaz were social FLE. These different patterns seemed to be dominant in the other timescales as well. However, it is worth noting that, despite the dominance of unique patterns of enjoyment in moments of each individual learner timescale, these patterns overlapped within the ecology of differing scales, rendering the two participants’ experiences similar in moments of enjoyment. For instance, the role of the teacher was really important as an attractor in affording “self–teacher” moments of enjoyment for both Sara and Sanaz.

## Conclusion

Following Dewaele and Li’s (2020) recommendation for researchers to develop original methodological approaches to explore the dynamic nature of FLE, in this study, we aimed to apply a potentially rich methodological approach, EMA, to investigate the dynamics of two foreign language learners’ moments of enjoyment. The results of this study indicated that the nature of each learner’s enjoyment moments is unique to that individual, which is susceptible to the main ecological drivers of change. On the other hand, the findings indicated that each ecological timescale contributes differently to the emergence of enjoyment patterns for each individual. Furthermore, despite the dominance of some unique patterns of enjoyment for each individual at different timescales, the two participants experienced similar patterns of enjoyment under the influence of the same ecological factors.

We hope that this study highlights the contribution of a complexity dynamic perspective to our insights into the dynamics of FLE in terms of its emergent dynamic patterns in different ecological timescales. The use of EMA in the exploration of the dynamics of FLE can be extended by future research via an event-based sampling scheme. However, we should note that understanding the dynamics of FLE in terms of its ecological temporal moments is still in its fledgling state. The exploration of these dynamics via EMA can shed lights on the situated nature of the FLE system. In general, building on the results of the current study and other related studies, we can gradually come up with a comprehensive model of FLE in terms of its dynamic nature. From a pedagogical perspective, the findings of this study indicate that even the same language learner might go through different levels of enjoyment in the different timescales of a course of language learning ranging from the micro scale of classroom interaction to the macro scale of the whole course. Teachers should notice that, despite the “self-”oriented determinants of their learners’ enjoyment, they can play a central role in their learners’ moments of high enjoyment. The importance of teachers cannot be underestimated, and their role increases through the creation of a positive and supportive atmosphere in their classes. On the other hand, teachers should be aware of the fact that the proportion of their role in the enjoyment moments of their students in not necessarily the same. Teachers should be especially attentive to the “self” factors underlying the private enjoyment zone of their students.

## Data Availability Statement

The datasets generated for this study are available on request to the corresponding author.

## Ethics Statement

The studies involving human participants were reviewed and approved by The Ethics Committee of the University of Bojnord. The patients/participants provided written informed consent to participate in this study.

## Author Contributions

All authors have participated in the data collection of the study and data analysis, helped to draft the manuscript, and read and approved final manuscript.

## Conflict of Interest

The authors declare that the research was conducted in the absence of any commercial or financial relationships that could be construed as a potential conflict of interest.
